# What is the response profile of deciduous pulp fibroblasts stimulated with *E. coli* LPS and *E. faecalis* LTA?

**DOI:** 10.1186/s12865-020-00367-8

**Published:** 2020-06-22

**Authors:** Bella Luna Colombini-Ishikiriama, Thiago Jose Dionisio, Thais Francini Garbieri, Rafaela Alves da Silva, Maria Aparecida Andrade Moreira Machado, Sandra Helena Penha de Oliveira, Vanessa Soares Lara, Andrew Seth Greene, Carlos Ferreira Santos

**Affiliations:** 1 Department of Biological Sciences, Bauru School of Dentistry, University of São Paulo, Bauru, SP Brazil; 2 Department of Surgery, Stomatology, Pathology and Radiology, Bauru School of Dentistry, University of São Paulo, Bauru, SP Brazil; 3 Department of Pediatric Dentistry, Orthodontics and Public Health, Bauru School of Dentistry, University of São Paulo, Bauru, SP Brazil; 4 Department of Basic Sciences, School of Dentistry of Araçatuba, São Paulo State University, Araçatuba, SP Brazil; 5 The Jackson Laboratory, Bar Harbor, ME USA

**Keywords:** Cytokines, Dental pulp, Fibroblasts and lipopolysaccharide

## Abstract

**Background:**

Oral fibroblast immunological responses to bacterial stimuli are well known. However, there are few studies about pulp fibroblasts from deciduous teeth (HDPF) responses, which are important for the treatment of pulp infections in children. The aim of this study was to evaluate expression and production of inflammatory cytokines and chemokines by HDPF when challenged with bacterial antigens normally present in advanced caries lesions.

**Methods:**

Triplicate HDPF from 4 children (*n* = 4; 2 boys and 2 girls) were cultured by explant technique and challenged or not with *Escherichia coli* lipopolysaccharide/1 μg/mL **(EcLPS)** or *Enterococcus faecalis* lipoteichoic acid/1 μg/mL **(EfLTA)** for 6 and 24 h. Most of published studies employed immortalized cells, i.e., without checking possible gender and genetic variables. mRNA expression and protein production were evaluated by RT-qPCR and ELISA MILLIPLEX®, respectively, for Interleukin (IL)-1α, IL-1β, IL-2, IL-4, IL-6, IL-8, IL-10, IL-12, IL-17, Chemokine C-C motif ligand 2/monocyte chemoattractant protein 1 (CCL2/MCP-1), Chemokine C-C motif ligand 3/macrophage inflammatory protein 1-alpha (CCL3/MIP1-α), Chemokine C-C motif ligand 5/ regulated on activation, normal T cell expressed and secreted (CCL5/RANTES), C-X-C motif chemokine 12/ stromal cell-derived factor 1 (CXCL12/SDF-1), Tumor Necrosis Factor-alpha (TNF-α), Interferon-gamma (IFN γ), Vascular Endothelial Growth Factor (VEGF), Colony stimulating factor 1 (CSF-1) and Macrophage colony-stimulating factor (M-CSF).

**Results:**

**EcLPS** increased IL-1α, IL-1β, IL-8, CCL2, CCL5, TNF-α and CSF-1 mRNA and protein levels while **EfLTA** was only able to positively regulate gene expression and protein production of IL-8.

**Conclusion:**

The results of the present study confirmed our hypothesis, since pulp fibroblasts from deciduous teeth are capable of increasing gene expression and protein production after being stimulated with **EcLPS** and **EfLTA.**

## Background

Pulp tissue of deciduous teeth is often exposed to bacterial challenges arising from untreated carious lesions. The products of proliferation and metabolic activity of biofilm microorganisms that colonize these lesions degrade the enamel and dentin mineralized tissues reaching the connective tissue located at the center of the tooth. During this process, proliferation and metabolic activity products of the microorganisms and dentin matrix bioactive molecules are released and defunds towards dentinal tubules starting host protective events including antibacterial, immune, and inflammatory responses against this bacterial invasion [1, 2].

After been recognized by odontoblasts cell layer by Toll-like receptor (TLR) family, the Pathogen-Associated Molecular Patterns (PAMPs), presented in its cell membrane, starts the effector phase of innate immune response [3, 4]. The activation of TLR upregulates innate immunity effectors, including production of antimicrobial agents and proinflammatory cytokines and chemokines [5, 6], with the objective of recruit and activate cells present in tissue resident and blood, to eliminate pathogens and allow repair [7, 8].

Fibroblasts are the most abundant cells in the pulp and can play immunoinflammatory roles after recognition of lipopolysaccharide (LPS) via TLR 4 and lipoteichoic acid (LTA) via TLR2. TLRs act as sentinel receptors and their stimulation leads to activation of transcription factors such as nuclear factor-k B and interferon regulatory factors, which promote the transcription of proinflammatory cytokines and other proteins that promote host defenses [9]. In humans, the are 11 different TLRs that recognize different molecules native to bacteria, viruses, fungi and protozoa [10, 11]. In view of the presence of TLRs in fibroblasts, stimuli with LPS from *Escherichia coli* and LTA from *Enterococcus faecalis* were used to ascertain the behavior of pulp fibroblasts from deciduous teeth.

Studies have shown that pulp tissues of inflamed permanent teeth have increased levels of various cytokines such as IL-1β [12, 13], IL-17 [14], Il-6 and IL-8 [13, 15, 16], TNF-α [17], MIP-3α [18] and CXCL10 [19]. Cultured fibroblasts derived from permanent pulp tissue also can produce cytokines/chemokines like IL-1β, IL-6, IL-8, IL-17, IL-23, CCL3, and CXCL12 when challenged by other cytokines [14, 20] or by microbial components [13, 16, 21–23].

However, studies of the expression/production of these cytokines and chemokines in pulp tissues or even in cultured cells of deciduous pulps are scarce in the literature. It is known that Human Deciduous Pulp Fibroblasts (HDPF) are capable of producing cytokines such as IL-1β and IL-8 against pulp capping materials [24], CCL3 and CXCL12 in response to stimulation with LPS and LTA [22, 23]. It is of fundamental importance to increase the knowledge on the role of fibroblasts in the expression/production of these molecules.

Our hypothesis is that, challenged with antigens, pulp fibroblasts from deciduous teeth may produce many other inflammatory cytokines. The purpose of the present study was to evaluate the expression and production of inflammatory cytokines and chemokines by HDPF when challenged by *Escherichia coli* lipopolysaccharide or *Enterococcus faecalis* lipoteichoic acid.

## Methods

### Cell culture

Human pulp fibroblasts were obtained from 6 teeth indicated for extraction due to orthodontic reasons, which were donated by 4 children (2 boys and 2 girls) aged 7–11 years. After their legal parents or guardians and children’s written consent, teeth were donated. Ethical approval was obtained from the Ethics Committee for Human Research of the Bauru School of Dentistry, University of São Paulo (CAAE: 44739015.0.0000.5417).

Pulp tissue was removed in aseptic conditions and cultured by using an explant technique as described previously [23]. Tissues were fragmented and incubated for cell growth in Dulbecco’s modified Eagle medium (DMEM) (Invitrogen, Life Technologies Corp, Carlsbad, CA) supplemented with 10% fetal bovine serum (FBS) (Gibco, Invitrogen, Carlsbad, CA) and antibiotics (100 mg/mL penicillin, 100 mg/mL streptomycin, 0.5 mg/mL amphotericin B; Invitrogen). Cultures were maintained at 37 °C in a humidified atmosphere of 5% CO_2_ and 95% air. Cells were used between the fourth and eighth passages.

### Cell viability

The concentrations of the antigens *Escherichia coli* lipopolysaccharide/1 μg/mL (EcLPS) or *Enterococcus faecalis* lipoteichoic acid/1 μg/mL (EfLTA) used in this study were previously tested about their toxicity under HDPF and did not show any effect in cell viability after 24 h of stimulus [23]. The phenotypic characterization of fibroblasts was carried out according to previously publication [23].

### Fibroblast stimulation

Cells were trypsinized and plated at a concentration of 5 × 10^4^ cells/well in 24-well plates. After 24 h to allow cellular attachment, medium alone (DMEM 1% FBS) **(Controls)** or containing *Escherichia coli* lipopolysaccharide/1 μg/mL **(EcLPS)** (L4391; Sigma-Aldrich, St Louis, MO) or *Enterococcus faecalis* lipoteichoic acid/1 μg/mL **(EfLTA)** (L4015; Sigma-Aldrich) was added to the cells in triplicate, according to our previous studies [21, 22, 25].

After the experimental times of 6 or 24 h, the supernatant and cells were collected and analyzed by enzyme-linked immunosorbent assay (ELISA MILLIPLEX®) and quantitative real-time polymerase chain reaction (qPCR), respectively.

### Real-time qPCR

Total RNA was obtained directly from cells using RNA-extraction columns kit (PureLink™ RNA Mini Kit, Ambion, Thermo Fisher Scientific, Waltham, MA) according to the manufacturer’s instructions. The quantity and purity of RNA extracted were analyzed using a spectrophotometer Nanodrop 1000 (Thermo Fisher Scientific). RNA was treated with DNAse (gDNA wipeout; Qiagen) and processed using the High Capacity cDNA Reverse Transcription Kit (Applied Biosystems, Thermo Fisher Scientific). qPCR was performed using the TaqMan system for qPCR and proprietary 20x FAM and MGB dye labeled probe-primer mix listed in Supplementary Table 1. All experiments were performed in a Real Time PCR System (Viia 7, Applied Biosystems, Thermo Fisher Scientific) using the comparative cycle threshold (Ct) method (ΔΔCt) as previously described [26]. mRNA expression of all the target genes were normalized to the RPL13A reference gene (Supplementary Table 1).

### Cytokine/chemokine detection in fibroblasts supernatants by ELISA MILLIPLEX®

ELISA MILLIPLEX**®** was performed to detect in supernatants of deciduous pulpal fibroblasts cultures the presence of 18 Cytokine/Chemokine, using the HCYTOMAG-60 K ELISA MILLIPLEX Kit (Cat.# HCYTOMAG-60 K, Millipore, SIGMA) according to the manufacturer’s protocol (Supplementary Table 2).

Briefly, 96-well plates were previously washed, in a shaker for 10 min (20-25 °C), with wash buffer solution (200 μL) before being dried and receiving 25 μL of standard or controls into appropriate wells and assay buffer (25 μL) to samples wells. Then, the same volume of DMEM (25 μL) was added to the background, standards and control wells and 25 μL of supernatants samples were added to the appropriate wells. In sequence, Mixing Bottle was vortexed and 25 μL of Mixed Beads (mix of 18 target beads) were added to the wells and incubated, under agitation on a plate shaker, wrapped with a foil, overnight at 4 °C.

After the incubation period, the well contents were gently removed and the plate was washed twice, with wash buffer solution (200 μL), under agitation for 30 s and the aid of a magnetic plate washer (60 s). Then, 25 μL of Detection Antibodies were added to the wells, and incubated, under agitation, wrapped with a foil, for 1 h at 20-25 °C.

The wells then received 25 μL of streptavidin-phycoerythrin and the plate was covered with a foil and incubated under agitation for 30 min at 20-25 °C. After this period, well contents were gently removed and the plate was washed twice, with wash buffer solution (200 μL), under agitation for 30 s and the aid of a magnetic plate washer (60 s). Finally, 150 μL of Sheath Fluid were added to all wells, the beads were resuspended on a plate shaker for 5 min and the plate ran on LUMINEX® 200™ for the detection of target cytokines/chemokines amounts in pg/mL.

### Statistical analysis

Statistical analysis was performed with GraphPad Prism 6.0 software (GraphPad Prism 6.0, GraphPad Software, San Diego, CA, USA). Since all data were parametric, a non-paired t test was used, observing the differences between the stimulated group in relation to the respective control in the same period of evaluation. Differences were identified using significance level set at 95% (*p* < 0.05).

## Results

### Phenotypic characterization of fibroblasts

**Fig. 1:**
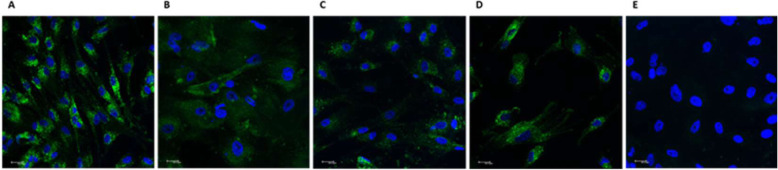
**-** Phenotypic characterization of HDPF by Fibroblast Surface Protein (FSP) staining. HDPF from 4 different patients (**A, B, C and D)** were plated (10^4^ cells/well) and showed positive staining for FSP-1 protein (green). Cell nuclei were stained with DAPI- blue (4′,6- diamidino-2-phenylindole dihydrochloride). **E** – negative control. Images captured by a confocal microscope (TCS model, SPE, Leica**®**, Mannheim, Germany). Scale bars - 20 μm

Deciduous Dental Pulp Fibroblasts were isolated and cultured as previously described [22, 23]. Positivity for Fibroblast Surface Protein- 1 (FSP-1) indicated their fibroblastic phenotype, as shown in Fig. 1.

### Cytokine/chemokine mRNA expression in deciduous pulp fibroblasts challenged by EcLPS and EfLTA

**Fig. 2:**
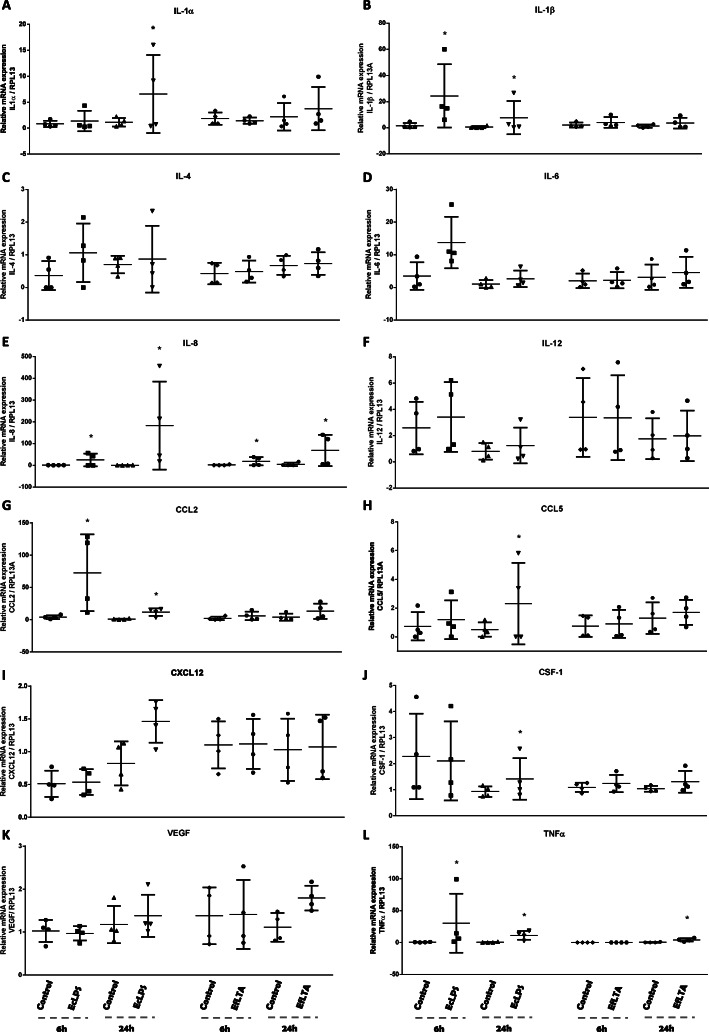
qPCR analysis of cytokines/chemokines by HDPF stimulated without **(Control)** or with lipopolysaccharide from *Escherichia coli***(EcLPS)** or *Enterococcus faecalis* lipoteichoic acid **(EfLTA)** (1 μg/mL- 6 and 24 h). Relative expression levels of the target mRNA relative to RPL13 mRNA from 4 donors in triplicate **(*n*** **= 4)** are displayed in graphs. **A**- Interleukin-1α (IL-1α); **B**-Interleukin-1β (IL-1β); **C**- Interleukin-4 (IL-4); **D**- Interleukin-6 (IL-6); **E**- Interleukin-8 (IL-8); **F**- Interleukin-12 (IL-12); **G-** Monocyte chemoattractant protein 1 (MCP-1/CCL2); **H**- RANTES (CCL5); **I**- SDF-1 (CXCL12); **J**- Colony-stimulating fator-1 (CSF-1); **K**- Vascular Endothelial Growth Factor (VEGF) and **L**- Tumor necrosis factor-α (TNF-α). The mean values observed for each patient are represented as a symbol and compare the stimulated values with their respective control group in the same experimental period, by non-paired t test. * indicates significant difference in relation to respective control in the same experimental period (*p* < 0.05)

mRNA evaluation of the cytokine/chemokine profile produced by HDPF challenged by EcLPS and EfLTA (1 μg/mL; 6 and 24 h) is described in Fig. 2.

Among the 18 targets studied, six of them were not expressed and did not suffer alteration in the expression when stimulated by both bacterial antigens – IL-2, IL-10, IL-17, IL-18, CCL3 and INF-λ **(data not show).**

However, 12 targets had mRNA expression detected in HDPF (Fig. 2) and the challenge generated by the presence of bacterial antigens showed a different behavior in the responses between EcLPS and EfLTA. EcLPS challenge was able to positively regulate the expression of the target genes of various cytokines studied - IL-1α, IL-1β, IL-8, CCL2, CCL5 TNF-α and CSF-1 (Fig. **2** A, B, E, G, H, J, L), whereas EfLTA was only able to positively regulate gene expression of IL-8 (Fig. 2E).

### Cytokine/chemokine secretion in supernatants of deciduous pulp fibroblasts challenged by EcLPS and EfLTA

**Fig. 3:**
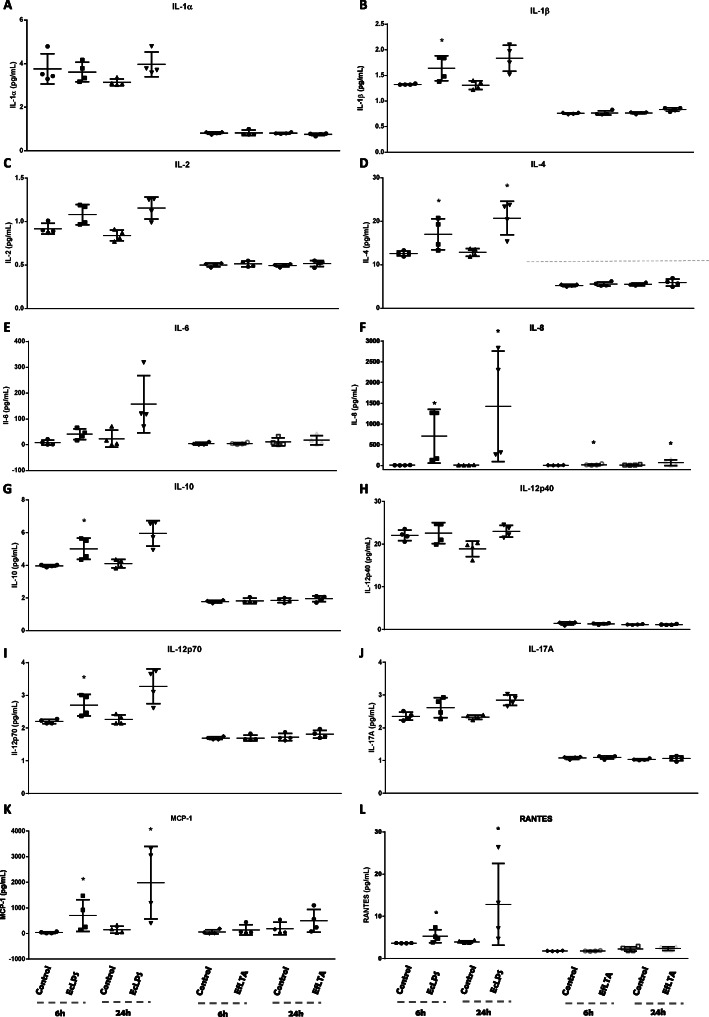
**– IL-1α (A), IL-1β (B), IL-2 (C), IL-4 (D), IL-6 (E), IL-8 (F), IL-10 (G), IL-12p40 (H), IL-12p70 (I), IL-17A (J), CCL2 (K) and CCL5 (L)** protein levels in HPFD supernatants challenged, or not **(Control),** with Lipopolysaccharide from *Escherichia coli***(EcLPS)** or *Enterococcus faecalis* lipoteichoic acid **(EfLTA**) for 6 and 24 h. ELISA MILLIPLEX® was performed and the mean of a triplicate of each patient (n = 4) was compared using non-pared t test. Each stimulated group was compared with their control in the same experimental period. * (p < 0.05)

**Fig. 4:**
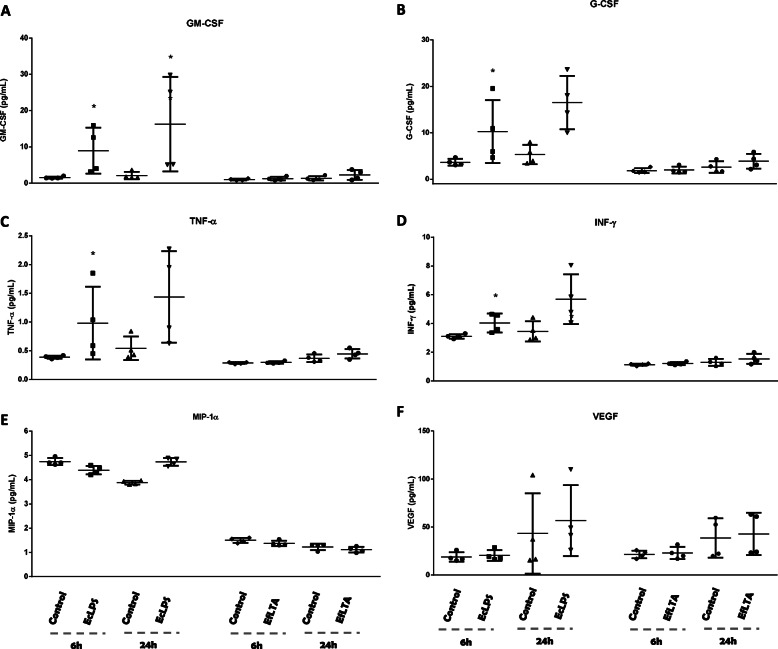
**– MCP-1 (A), RANTES (B), GM-CSF (C), CSF (D), TNF-α (E), INF-λ (F), MIP-1α (G), VEGF (H)** protein levels in HPFD supernatants challenged, or not **(Control),** with Lipopolysaccharide from *Escherichia coli***(EcLPS)** or *Enterococcus faecalis* lipoteichoic acid **(EfLTA**) for 6 and 24 h. ELISA MILLIPLEX® was performed and the mean of a triplicate of each patient (n = 4) was compared using non-pared t test. Each stimulated group was compared with the control in the same experimental period. * (p < 0.05)

The same behavior observed in the evaluation of mRNA expression for cytokines/chemokines was observed when evaluating the protein presence in the supernatants of HPFD cultures stimulated or not by EcLPS and EfLTA. Lipopolysaccharide from *Escherichia coli* statistically stimulated the production of some cytokines and chemokines, being more evident in the evaluation period of 6 h (IL-1β, IL-4, IL-8, IL-10, IL-12p70 **(**Fig. 3**– B, D, F, G, H),** G-CSF, GM-CSF and INF-λ **(**Fig. 4**- C, D, F)** as compared to the 24-h period (IL-4, IL-8 and GM-CSF) **(**Figs. 3B, F and 4C**)**. EfLTA also was only capable to up-regulate the production of IL-8, in both experimental periods (Fig. 3F).

## Discussion

Immune inflammatory response of pulp tissue from permanent teeth to the installation and progression of carious lesions has been studied over the last years. Among the events involved in this response against the various pathogens, it has been reported the importance of the upregulation of proinflammatory cytokines/chemokines and other inflammatory mediators, which are responsible to recruit and activate tissue resident and blood borne immune/inflammatory cells [2, 7, 8]. However, little is known about the production of such mediators and their role in the pulpal response of deciduous teeth.

In the present study, we were able to detect an upregulation in mRNA expression and protein production, by fibroblasts from primary teeth of the four different donors, of various cytokines/chemokines when these cells were challenged by EcLPS, while EfLTA only positively regulated IL-8 expression and production. These results are in agreement with the few works in the literature that have studied the production of cytokines and chemokines by these cells [22–24]. Sipert et al. and Ferreira et al. [23, 24] observed the upregulation of CCL3 and IL-8, respectively, only when EcLPS was tested. These cells type were also stimulated by PgLPS (*Porphyromonas gingivalis* lipopolysaccharide) to upregulate CCL3 production, but not CXCL12. These results reflect the different capacities of these virulence factors to initiate or maintain immune responses by deciduous pulp fibroblasts and show that although less intensely than LPS, EfLTA is able to induce HDPF to release IL-8, thus suggesting a possible participation in pulpal disease establishment or maintenance. LPS and LTA have different molecular structures and particular antigenic portions that result in different recognition pattern which will generate the specific response against that bacteria [27, 28].

Two factors are important to observe when evaluating the response profile to EcLPS challenge. The first one concerns the variation in the production intensity of the cytokines/chemokines studied. In terms of the amount produced it was possible to observe, distinct groups, despite the significant difference observed for all (*p* < 0.05). In the group in which the detected production was most intensely stimulated by EcLPS are CCL2 and IL-8, followed by IL-6. In another group in which this production was less intense are IL-4, G-CFS, GM-CSF and CCL5, followed by a group where they are detected in very low amounts (IL-1β, IL-10, IL-12p70, IL-17A, TNF-α and INF-λ).

Although the production of most of these cytokines/chemokines by HPFD has never been studied before, the few existing studies have observed both similar and different results from those obtained in our study. While a significant increase in the production of MIP-1α/CCL3 and a decrease of CXCL12 by these cells have been observed, these variations were not detected by our study [22, 23]. Ferreira et al. 2009, as well as in our study, also detected, in supernatants from HPFD cultures challenged with EcLPS, a significant increase in the production of IL-1β and IL-8, when used as a control stimulus [24]. It is noteworthy that, while the other studies use cells from only one donor, our study is the only one that evaluates the production of cytokines by cells of four different donors, two boys and two girls, which considerably increases the reliability of the results presented, despite the expected variability among individuals.

The second concerns the kinetics of the production of these mediators. We observed that among the 18 cytokines/chemokines evaluated, 11 had their production stimulated by EcLPS mainly in the period of 6 h of stimulus. Of these, only five maintained high levels of production after 24 h of stimulation. Sipert et al. and Ferreira et al. [23, 24] observed some similar and some different results as compared to ours, being such differences related to the EcLPS concentrations tested and cytokines evaluated. This kinetic evaluation is extremely important since it allows to identify some kind of immune response pattern against the EcLPS by this cell type, never described before, and can collaborate in the process of understanding the role of these molecules produced by these fibroblasts in combating bacterial invasions of the deciduous teeth pulp.

Several cytokines had their production stimulated by EcLPS used in our study, and the role of each of them should be further evaluated. However, MCP-1/CCL2, IL-8 and IL-6 had higher levels in comparison to the others. Although increased amounts of these molecules have already been identified in pulps of inflamed permanent teeth and cultures of pulp fibroblasts of permanent teeth stimulated by other cytokines, bacterial antigens or dental materials their role in the response of deciduous pulps remains unknown [13–16]. Monocyte chemoattractant protein-1 (MCP-1/CCL2), IL-8 and IL-6 are key chemokines that regulate activation, migration and infiltration of monocytes/macrophages, neutrophils and T lymphocytes from the bloodstream across the vascular endothelium required to inflammation [2, 29] and its influence on cell migration and angiogenesis can lead to higher regenerative potential of dental pulp [2, 30].

The results observed in our study may demonstrate the great potential that pulp fibroblasts of deciduous teeth have to contribute rapidly to the recruitment of neutrophils and lymphocytes from the vessels to the tissues affected by bacteria. Therefore, further studies using different antigens and other experimental conditions should be carried out in order to better understand the ability of HDPF to collaborate with the immune inflammatory response of the pulp against microorganisms.

## Conclusion

The results of the present study confirmed our hypothesis, since pulp fibroblasts from deciduous teeth are capable of increasing gene expression and protein production after being stimulated with EcLPS and EfLTA.

## Supplementary information

**Additional file 1: Table 1.** Catalog numbers of inventoried PCR assays (Applied Biosystems, USA).

**Additional file 2: Table 2.** Cytokine/Chemokine detected by HCYTOMAG-60 K MILLIPLEX® Kit.

## Data Availability

The datasets used and/or analyzed during the current study are available from the corresponding author on reasonable request.

## Abbreviations

CCL2/MCP-1: Chemokine C-C motif ligand 2/monocyte chemoattractant protein 1
CCL3/MIP1-α: Chemokine C-C motif ligand 3/macrophage inflammatory protein 1-alpha
CCL5/RANTES: Chemokine C-C motif ligand 5
CSF-1: Colony stimulating factor 1
CXCL12/SDF-1: C-X-C motif chemokine 12/ stromal cell-derived factor 1
DMEM: Dulbecco’s modified Eagle medium
EcLPS: *Escherichia coli* Lipopolysaccharide
EfLTA: *Enterococcus faecalis* Lipoteichoic Acid
FAPESP: State of São Paulo Research Foundation
FBS: Fetal Bovine Serum
FSP-1: Fibroblast Surface Protein- 1
HDPF: Human Deciduous Pulp Fibroblasts
IFN γ: Interferon-gamma
IL: Interleukin
LPS: Lipopolysaccharide
LTA: Lipoteichoic acid
M-CSF: Macrophage colony-stimulating factor
PAMPs: Pathogen-Associated Molecular Patterns
qPCR: Quantitative real-time polymerase chain reaction
TLR: Toll-like receptor
TNF-α: Tumor Necrosis Factor-alpha
VEGF: Vascular Endothelial Growth Factor
