# Supplementary material for: What is the response profile of deciduous pulp fibroblasts stimulated with E. coli LPS and E. faecalis LTA?
Source: BMC Immunol. 2020 Jun 22;21:38. doi: 10.1186/s12865-020-00367-8 (PMC7310245; doi:10.1186/s12865-020-00367-8)
Supplement: Supplementary file 1 — Additional file 1: Table 1. Catalog numbers of inventoried PCR assays (Applied Biosystems, USA). [file 12865_2020_367_MOESM1_ESM.docx]

**Table 1.** Catalog numbers of inventoried PCR assays (Applied Biosystems, USA).

| **TARGET** | **CATALOG NUMBER** |
| --- | --- |
| Interleukin-1α (IL-1α) | Hs00174092_m1 |
| Interleukin-1β (IL-1β) | Hs01555410_m1 |
| Interleukin-2 (IL-2) | Hs00174114_m1 |
| Interleukin-4 (IL-4) | Hs00174122_m1 |
| Interleukin-6 (IL-6) | Hs00174131_m1 |
| Interleukin-8 (IL-8) | Hs00174103_m1 |
| Interleukin-10 (IL-10) | Hs00961622_m1 |
| Interleukin-12 (IL-12) | Hs01073447_m1 |
| Interleukin-17 (IL-17) | Hs 00174383_m1 |
| Monocyte chemoattractant protein 1 (MCP-1/CCL2) | Hs00234140_m1 |
| MIP-1α (CCL3) | Hs00234142_m1 |
| RANTES (CCL5) | Hs 00982282_m1 |
| SDF-1 (CXCL12) | Hs03676656_m1 |
| Tumor necrosis factor-α (TNF-α) | Hs00174128_m1 |
| Interferon-γ (IFNγ) | Hs00989291_m1 |
| Vascular Endothelial Growth Factor (VEGF) | Hs 00900055_m1 |
| Colony-stimulating factor-1 (CSF-1) | Hs00174164_m1 |
| RPL13A | Hs01578912_m1 |
