# Supplementary material for: What is the response profile of deciduous pulp fibroblasts stimulated with E. coli LPS and E. faecalis LTA?
Source: BMC Immunol. 2020 Jun 22;21:38. doi: 10.1186/s12865-020-00367-8 (PMC7310245; doi:10.1186/s12865-020-00367-8)
Supplement: Supplementary file 2 — Additional file 2: Table 2. Cytokine/Chemokine detected by HCYTOMAG-60 K MILLIPLEX® Kit. [file 12865_2020_367_MOESM2_ESM.docx]

**Table 2.** Cytokine/Chemokine detected by HCYTOMAG-60K MILLIPLEX^®^ Kit.

| **TARGET** |
| --- |
| Interleukin-1α (IL-1α) |
| Interleukin-1β (IL-1β) |
| Interleukin-2 (IL-2) |
| Interleukin-4 (IL-4) |
| Interleukin-6 (IL-6) |
| Interleukin-8 (IL-8) |
| Interleukin-10 (IL-10) |
| Interleukin-12p40 (IL-12p40) |
| Interleukin-12p70 (IL-12p70) |
| Interleukin-17A (IL-17A) |
| Monocyte chemoattractant protein 1 (MCP-1/CCL2) |
| MIP-1α (CCL3) |
| RANTES (CCL5) |
| Tumor necrosis factor-α (TNF-α) |
| Interferon-γ (IFNγ) |
| Vascular Endothelial Growth Factor (VEGF) |
| Colony-stimulating factor-1 (CSF-1) |
| Macrophage Colony-stimulating fator-1 (MCSF-1) |
